# Neuroinflammation, Blood–Brain Barrier, and HIV Reservoirs in the CNS: An In-Depth Exploration of Latency Mechanisms and Emerging Therapeutic Strategies

**DOI:** 10.3390/v17040572

**Published:** 2025-04-16

**Authors:** Noor Said, Vishwanath Venketaraman

**Affiliations:** College of Osteopathic Medicine of the Pacific, Western University of Health Sciences, Pomona, CA 91766-1854, USA; noor.said@westernu.edu

**Keywords:** HIV, reservoirs, HAND, blood–brain barrier, drug delivery, latency-reversing agents, gene editing, nanoparticles, inflammasome

## Abstract

Despite the success of antiretroviral therapy (ART) in suppressing viral replication in the blood, HIV persists in the central nervous system (CNS) and causes chronic neurocognitive impairment, a hallmark of HIV-associated neurocognitive disorders (HAND). This review looks at the complex interactions among HIV, the blood–brain barrier (BBB), neuroinflammation, and the roles of viral proteins, immune cell trafficking, and pro-inflammatory mediators in establishing and maintaining latent viral reservoirs in the CNS, particularly microglia and astrocytes. Key findings show disruption of the BBB, monocyte infiltration, and activation of CNS-resident cells by HIV proteins like Tat and gp120, contributing to the neuroinflammatory environment and neuronal damage. Advances in epigenetic regulation of latency have identified targets like histone modifications and DNA methylation, and new therapeutic strategies like latency-reversing agents (LRAs), gene editing (CRISPR/Cas9), and nanoparticle-based drug delivery also offer hope. While we have made significant progress in understanding the molecular basis of HIV persistence in the CNS, overcoming the challenges of BBB penetration and neuroinflammation is key to developing effective therapies. Further research into combination therapies and novel drug delivery systems will help improve outcomes for HAND patients and bring us closer to a functional cure for HIV.

## 1. Introduction

Antiretroviral therapy (ART) has changed the face of Human Immunodeficiency Virus (HIV) management, reducing viral loads and increasing life expectancy. However, the central nervous system (CNS) is a sanctuary site for HIV, harboring latent viral reservoirs that evade the immune system and ART [1]. HIV in the CNS is the cause of HIV-associated neurocognitive disorders (HANDs), a spectrum of cognitive impairment from mild dysfunction to severe dementia [2]. The underlying mechanisms of HANDs include chronic neuroinflammation, neuronal injury, and synaptic dysfunction [3]. HANDs affect approximately 30–50% of people living with HIV, even in the era of effective ART, highlighting the urgent need for therapies and interventions that can penetrate the blood–brain barrier (BBB) and target latent viral reservoirs in the CNS [4].

HIV accesses the CNS through infected monocytes that cross the BBB [5]. Once in the CNS, the virus establishes reservoirs in microglia and astrocytes, using cellular and molecular mechanisms to maintain latency [1]. Disruption of the BBB, driven by viral proteins like Tat and gp120, allows for viral persistence and amplifies the neuroinflammation [6]. This inflammatory environment, with activation of the NLRP3 inflammasome and release of pro-inflammatory cytokines, promotes the chronic neuroinflammatory state, exacerbating neuronal damage and cognitive decline [7].

The presence of HIV in the CNS is a major hurdle to a cure, primarily due to HIV latency, a state where the virus is dormant in infected cells and hidden from immune surveillance in the CNS [8]. Understanding the mechanisms by which HIV establishes and maintains latency in the CNS is crucial to developing ways to target and eliminate these reservoirs. The disruption of the BBB, a major protector of the CNS, is key to HIV migrating into the brain and supporting viral persistence [9].

This review will cover the current understanding of HIV latency in the CNS, the role of the BBB in HIV pathogenesis, and the latest strategies to target latent HIV reservoirs. We combine recent findings on epigenetics, new drug delivery, and latency reversal to show the way to better treatments for HAND and a functional cure. By understanding the molecular, cellular, and systemic factors that contribute to HIV persistence in the brain, we can think about potential treatments. This review highlights the need for combination approaches and new delivery systems to achieve a functional cure and improved outcomes for people with HAND.

## 2. HIV and BBB Disruption

A major barrier to curing HIV from the CNS is the BBB, a selective barrier that regulates what gets into the brain from the blood [10]. HIV can break the BBB and cause neurological problems. The BBB is compromised in HIV infection, creating a way for the virus to get in and for viral reservoirs to be established in the brain [11]. This is multifactorial in both the direct effects of the virus and the immune response it triggers.

**Figure 1:**
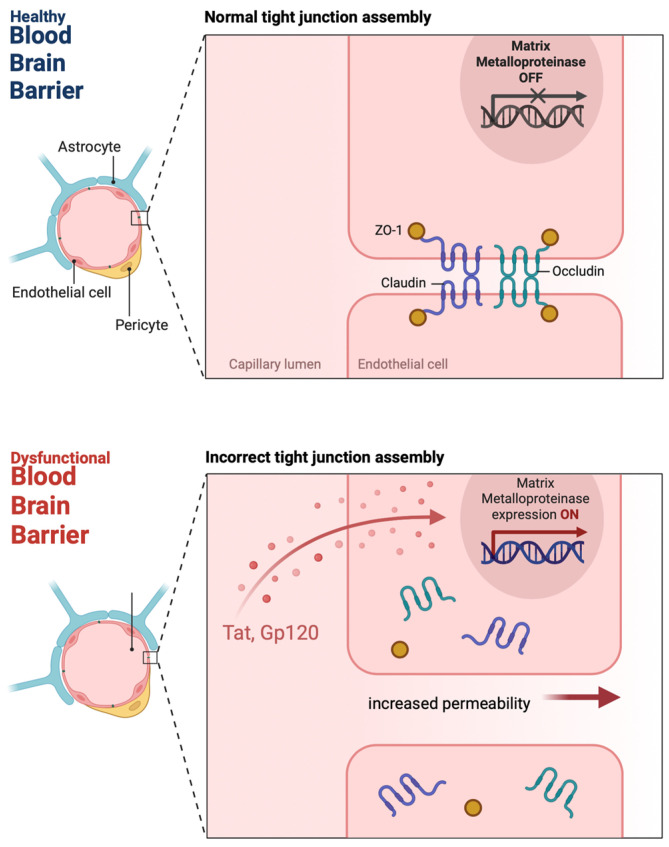
Increased permeability of the BBB due to the upregulation of MMPs. Tat and gp120 both disrupt BBB through oxidative stress, apoptosis, and tight junction protein disruption, allowing HIV to enter the CNS and cause neurocognitive disorders. Tat, a regulatory protein of HIV, upregulates matrix metalloproteinases (MMPs), enzymes that break down the extracellular matrix, and tight junction proteins that normally keep the BBB intact. As a result, the BBB becomes more permeable, and immune cells and other pathogens can infiltrate, perpetuating the cycle of neuroinflammation and viral spread.

BBB disruption in HIV infection involves multiple pathways and cell types. The HIV-1 envelope glycoprotein (gp), gp120, has been shown to compromise BBB integrity by altering tight junction proteins in human brain microvascular endothelial cells (HBMECs) [12] (Figure 1). This increases BBB permeability and allows monocytes to cross the BBB through PKC and calcium release-mediated pathways. Gp120 causes oxidative stress in brain endothelial cells by decreasing antioxidants and increasing oxidative stress markers [13]. Gp120 can bind to chemokine receptors on endothelial cells and activate signaling pathways that increase BBB permeability. This protein also allows infected monocytes to transmigrate across the BBB and enter the CNS. Gp120-induced BBB dysfunction involves the activation of protein kinase C (PKC) pathways and receptor-mediated calcium release, leading to cytoskeletal changes, endothelial cell junctions, and monocyte migration [12,13].

Trans-activator of transcription (Tat), a regulatory protein of HIV, upregulates matrix metalloproteinases (MMPs), enzymes that break down the extracellular matrix, and tight junction proteins that normally keep the BBB intact [14] (Figure 1). As a result, the BBB becomes more permeable, and immune cells and other pathogens can infiltrate, perpetuating the cycle of neuroinflammation and viral spread [15]. Tat also causes oxidative stress in brain endothelial cells, leading to a decrease in intracellular antioxidants like glutathione and an increase in oxidative markers like malondialdehyde [16]. Tat also induces apoptosis in human brain microvascular endothelial cells by activating nitric oxide synthase (NOS) pathways, which increases the permeability of BBB [17]. Tat also decreases the expression of tight junction proteins like occludin and zonula occludens (ZO)-1 and ZO-2 partly by activating MMP-9 [15,18]. Additionally, Tat affects focal adhesion assembly and cytoskeletal organization in HBMECs, further compromising the BBB integrity [13,15,17]. In summary, Tat and gp120 both disrupt BBB through oxidative stress, apoptosis, and tight junction protein disruption, allowing HIV to enter the CNS and cause neurocognitive disorders.

HIV infection of astrocytes, although minimal, can also compromise BBB integrity. This is through gap junction-dependent mechanisms leading to endothelial apoptosis and dysregulation of various signaling pathways in astrocytes [19]. Also, HIV-1 infection of HBMECs activates pro-inflammatory and interferon-inducible genes, contributing to BBB dysfunction through inflammation and cytokine signaling [20]. HIV-1 infected monocytes can cross the BBB more efficiently, which is further facilitated by systemic lipopolysaccharide (LPS) levels that compromise the BBB integrity [21,22]. BBB disruption in HIV is complex and involves viral, immune cells, and inflammatory factors, leading to permeability and neurocognitive problems.

In addition to direct viral effects, the inflammatory response to HIV itself is a major contributor to BBB dysfunction. Prolonged elevation of pro-inflammatory cytokines like IL-6, TNF-α, and interferons increases BBB permeability and leukocyte adhesion and infiltration into the brain [19]. This low-grade chronic inflammation creates a feedback loop, amplifying both viral replication and immune cell trafficking to the CNS [23]. We now know this chronic neuroinflammation is a major contributor to long-term cognitive impairment in HIV-infected individuals.

While primary HBMEC models are useful for studying BBB integrity, they lack the full cellular architecture and dynamic immune surveillance in vivo. We need more animal models that mimic HIV neuropathogenesis, like humanized mice or simian immunodeficiency virus (SIV) infected macaques, to reinforce these findings in a physiological setting [24].

## 3. Monocyte Trafficking and HIV Entry into the CNS

One of the ways HIV gets into the CNS is through the trafficking of peripheral monocytes, especially the CD14+CD16+ subset, which are more likely to carry the virus across the BBB [25]. Under normal conditions, monocytes migrate into the brain in response to inflammation or infection, but in the case of HIV, these immune cells can be vehicles for viral dissemination [26]. Once in the brain, these infected monocytes become macrophages or infect resident microglia, the brain’s resident immune cells, making these monocytes a key target for therapeutic interventions [27,28].

Viral dynamics in monocytes demonstrate that the virus declines more slowly in monocytes than in activated CD4+ T cells [29]. The average half-life of viral DNA in monocytes/macrophages is longer than in both activated and resting CD4+ T cells, so monocytes/macrophages play a key role in ongoing replication in HIV-1 patients on combination ART (cART) [29]. In naive patients, activated CD4+ T cells account for nearly all (99%) of plasma viremia, and the remaining 1% is likely from tissue macrophages [30]. In the presence of cART, however, macrophages are likely the main source of plasma viremia as active viral replication in CD4+ T cells is suppressed [30,31].

Recent studies have shown the inflammatory signals that drive monocyte migration into the CNS [32]. High levels of chemokines, especially C-C chemokine ligand-2 (CCL2), are found in the brain and cerebrospinal fluid of HIV encephalitis patients, suggesting they play a key role in recruiting monocytes and HIV-infected leukocytes to the CNS [32,33]. Research on brain microvascular endothelial cells (BMVECs) shows that HIV accesses the brain when HIV-infected leukocytes cross the BBB through a CCL2-dependent mechanism during early HIV infection [33]. As per previous research, CCL2 levels are elevated in the cerebrospinal fluid (CSF) of people with HIV-associated dementia (HAD), which is the primary chemoattractant for leukocyte migration into the brain [33,34]. However, in the era of ART, serum CCL2 levels do not rise in aviremic patients, whereas viremic patients have significantly higher CCL2 levels in their serum [35]. This suggests that for patients with HIV on ART who are diagnosed with HAND, there may be an alternative pathway for leukocyte entry into the brain that is independent of both CCL2 and HIV replication [36].

Integrin-mediated adhesion and interaction with endothelial cells also play a big role in this process, allowing infected monocytes to transmigrate across the BBB, with molecules like CCR2, JAM-A, and ALCAM upregulated on HIV-infected CD14+CD16+ monocytes and allowing transmigration across the BBB [37]. Studies show that blocking these molecules with specific antibodies or CCR2/CCR5 dual inhibitors, like Cenicriviroc, reduces monocyte transmigration and is a promising therapeutic option to prevent CNS viral seeding and neuroinflammation [38,39]. HIV viral proteins, particularly Tat and gp120, have been shown to increase monocyte transmigration by upregulating CXCR3 and disrupting BBB integrity [14]. Tat and gp120 can interact with chemokine receptors and enhance monocyte trafficking, a mechanism for persistent neuroinflammation in HAND. These findings show the complex interplay among viral proteins, chemokine signaling, and monocyte trafficking in the context of HIV neuropathogenesis [14].

There are contradictory findings on the role of CCR2. Some studies show that CCR2 is required for monocyte entry into CNS, while others show that CCR5 plays a compensatory role, especially in chronic infection [40]. Other studies show that blocking CCR2 does not completely prevent monocyte migration, suggesting that alternative pathways must be involved [41]. There also seems to be methodological variability in chemokine analysis. For example, different methods used to measure chemokine expression (e.g., ELISA versus RNA sequencing versus flow cytometry) contribute to the variability in reported levels of CCL2 and other chemokines [11]. We need standardized methods to quantify chemokine signaling in CNS HIV infection. Further, most of the mechanistic studies on monocyte transmigration have been conducted in vitro, where BBB permeability and immune cell interactions may not fully capture the complexity of the living brain.

Blocking monocyte migration seems to be a good strategy, but we have little data on the long-term consequences of such interventions. Monocytes are essential for neuroimmune homeostasis, tissue repair, and clearance of cellular debris [42]. They differentiate into macrophages and dendritic cells and play a crucial role in maintaining cellular homeostasis, especially during infection and inflammation [43]. Prolonged monocyte trafficking inhibition may have unintended neurodegenerative consequences, as they guide vascular remodeling, stimulate local stem and progenitor cells, and facilitate the structural repair of tissues like muscle and bone [44,45]. Given these roles, any therapeutic approach to modulate monocyte activity must consider the potential for unintended neurodegenerative effects.

## 4. Chronic Microglial Activation, Neuroinflammation, and Neuronal Injury

HIV-associated neurocognitive disorders are a spectrum of cognitive impairment that occurs in HIV-infected individuals despite ART [46]. Neuronal injury in HAND is multifactorial, driven by both direct viral effects and the inflammatory environment. Neuronal injury and cognitive decline in HAND happen through a complex process involving chronic microglial activation, NLRP3 inflammasome activation, and disruption of the BBB by HIV-1 proteins Tat and gp120 [47].

**Figure 2:**
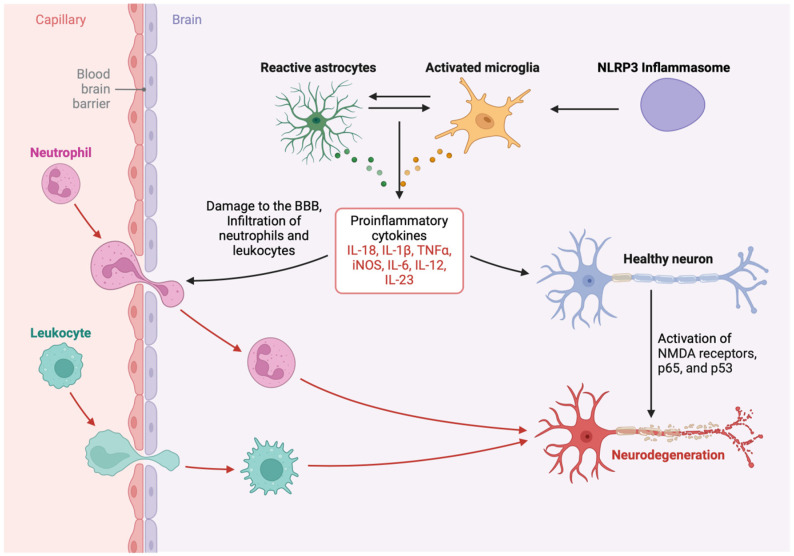
Role of astrocytes and microglia in neurodegeneration. The NLRP3 inflammasome plays a key role in microglial activation and the release of pro-inflammatory cytokines. The release of IL-18, IL-1β, TNF-α, and IL-6 from activated microglia worsens neuroinflammation and contributes to the pathogenesis of HAND. These cytokines create a pro-inflammatory environment that contributes to neuronal injury and death and further impairs cognitive function.

Microglia are the brain’s immune cells and are the first to respond to an HIV infection [48]. Upon encountering HIV or HIV proteins, microglia become activated and release pro-inflammatory cytokines and chemokines [49]. This activation creates a chronic inflammatory environment in the brain that is maladaptive for neuronal health. Microglia, the resident immune cells of the CNS, are the key players in HIV-associated neuroinflammation [50]. Upon activation by HIV proteins [Tat, gp120, viral protein R (Vpr)], microglia release a cascade of pro-inflammatory cytokines, including tumor necrosis factor-alpha (TNF-α), interleukin (IL)-1β, IL-6, and IL-18, which further amplify neuroinflammation and contribute to neuronal damage and neurocognitive disorders [20,51] (Figure 2). The activation of the NLRP3 inflammasome in microglia is a critical step in this process, as it facilitates this microglial activation and the maturation of these cytokines [20,21] (Figure 2). This chronic activation of microglia is thought to be one of the major contributors to neuronal damage and cognitive impairment in the HAND [52]. The release of glutamate by activated microglia can also cause excitotoxicity, a process where excessive glutamate causes neuronal injury and synaptic dysfunction and further contributes to cognitive decline [53]. This environment leads to oxidative stress, excitotoxicity, and direct cytokine-mediated neuronal damage, contributing to synaptic dysfunction, dendritic pruning, and neuronal apoptosis [51,52,54].

Despite recent studies, there are still many gaps in our understanding of how chronic microglial activation leads to HAND. While cross-sectional studies show increased microglial activation in HAND patients, there are no longitudinal studies tracking microglial activation over time. This leads to inquiring if microglial activation precedes cognitive decline or follows HIV-induced neurotoxicity. Also, many studies use different markers for microglial activation (e.g., Iba1, CD68, TSPO PET imaging), leading to inconsistent results [55]. Standardized criteria for microglial activation in HAND are needed to improve reproducibility across studies [56].

There is also a limited focus on neuroprotective mechanisms. Most studies focus on the neurotoxic effects of microglial activation, but few look at compensatory neuroprotective mechanisms [57]. For example, M2-polarized microglia have anti-inflammatory properties and promote neuronal repair, posing the question of whether we can target M2 polarization to mitigate HAND-related neuroinflammation [58].

We also see inconsistent evidence of glutamate excitotoxicity. While microglial activation releases glutamate, some studies suggest that neuronal glutamate receptor dysfunction rather than excess glutamate itself is the primary driver of excitotoxicity [59,60]. More research is needed on glutamate homeostasis in HAND.

## 5. NLRP3 Inflammasome Activation

The inflammasome complex, and especially the NLRP3 inflammasome, has been shown to play a role in microglial activation and the release of pro-inflammatory cytokines [61]. The NLRP3 inflammasome is a multi-protein complex that plays a key role in the innate immune response by activating caspase-1, which in turn cleaves and releases pro-inflammatory cytokines IL-1β and IL-18 [62]. In the context of HIV infection, the NLRP3 inflammasome is heavily implicated in microglial activation and neuroinflammation [62]. The NLRP3 inflammasome is key to microglial chronic activation, neuronal damage, and cognitive impairment in HAND.

The functioning of the NLRP3 inflammasome involves several key phases. The first stage, priming, includes the upregulation of NLRP3 and pro-IL-1β through the activation of the NF-κB signaling pathway [63]. Proteins from HIV-1, like Tat and gp120, can prime the NLRP3 inflammasome in microglial cells. For example, HIV-1 Tat has been demonstrated to induce NLRP3 expression in a manner that is both dose- and time-dependent, leading to increased levels of active caspase-1 and IL-1β, each a marker of the inflammasome activation [64].

The second signal involves the formation of the NLRP3 inflammasome complex, which consists of NLRP3, ASC (apoptosis-associated speck-like protein containing a CARD), and pro-caspase-1. This assembly is frequently initiated by mitochondrial reactive oxygen species (ROS) and other danger signals. Methamphetamine (Meth) use can exacerbate this mechanism by intensifying gp120-mediated NLRP3 activation, increasing ROS levels, and promoting the release of IL-1β and other pro-inflammatory substances [65].

The assembled inflammasome activates caspase-1, which subsequently cleaves pro-IL-1β into its active form, IL-1β, and processes gasdermin D (GSDMD) to induce pyroptosis, a type of inflammatory cell death [66]. This mechanism contributes to the neuroinflammatory milieu seen in HAND [67]. Tat and Vpr also help activate and assemble the inflammasome complex and induce the caspase-1 cleavage of pro-IL-1β into IL-1β, promoting neuroinflammation and neurobehavioral deficits [62]. The activation of the NLRP3 inflammasome in microglial cells results in the production of pro-inflammatory cytokines such as IL-1β and IL-18, which play a role in neuroinflammation and neuronal damage. Chronic treatment with NLRP3 inhibitors like MCC950 has been shown to reduce neuroinflammation and neuronal injury in gp120 transgenic mice, signaling a potential therapeutic target for HAND [67,68].

**Figure 3:**
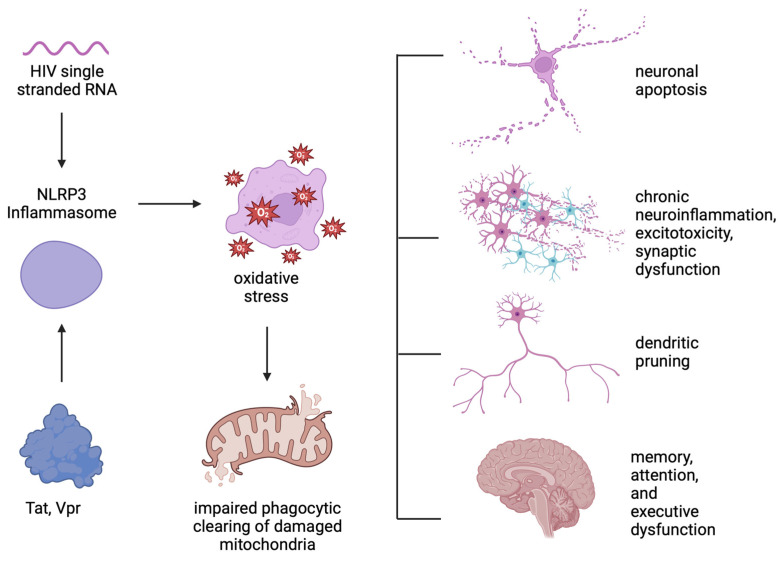
Effects of chronic activation of the NLRP3 inflammasome. HIV-1 single-stranded RNA (ssRNA40) and proteins such as Tat and Vpr activate the NLRP3 inflammasome in microglia, leading to the release of pro-inflammatory cytokines and ROS. The increase in oxidative stress impairs the autophagic clearance of damaged mitochondria and further exacerbates neuroinflammation and neurotoxicity, causing apoptosis, excitotoxicity, direct neuronal damage, synaptic dysfunction, and dendritic pruning, all of which result in cognitive impairment and memory, attention, and executive function deficits characteristic of HAND.

HIV-1 single-stranded RNA (ssRNA40) also activates the NLRP3 inflammasome in microglia, leading to the release of pro-inflammatory cytokines IL-1β, IL-18, TNF-α, and IL-1α [69]. This activation increases ROS, impairs the autophagic clearance of damaged mitochondria, and further exacerbates neuroinflammation and neurotoxicity [69,70] (Figure 3).

Chronic activation of the NLRP3 inflammasome in microglia leads to sustained release of pro-inflammatory cytokines and a persistent inflammatory environment [61]. This chronic neuroinflammation leads to oxidative stress, excitotoxicity, direct neuronal damage and synaptic dysfunction, dendritic pruning, and neuronal apoptosis [71], all of which result in cognitive impairment and memory, attention, and executive function deficits characteristic of HAND (Figure 3). 

While NLRP3 has been studied extensively in cultured microglia, showing strong mechanistic evidence for NLRP3 in HAND, few animal models or human post-mortem studies have been conducted to validate it in a physiological context. There are also unclear inflammasome-independent mechanisms. NLRP3 is a major player in HAND, but other inflammasomes like AIM2 and NLRC4 might also be involved in neuroinflammation [72,73]. Studies have shown that AIM2 and NLRC4 also contribute to brain injury in ischemic conditions, so they potentially could be involved in HAND [74].

The studies surrounding the NLRP3 inflammasome also use variable activation markers. Some studies use caspase-1 cleavage and IL-1β release as surrogate markers of inflammasome activation, but these can be influenced by other inflammatory pathways [74,75]. We need specific and quantitative biomarkers of NLRP3 activation in HAND.

There also happen to be contradictory results on microglia versus astrocytes. Microglia are considered the primary inflammasome-activating cells, but recent studies show that astrocytes can also produce IL-1β and contribute to neuroinflammation, raising the question of their overall contribution [76,77].

## 6. HIV Latency Mechanisms in the CNS

HIV persists in the CNS because the virus can establish latent reservoirs in various cell types, including microglia and astrocytes [1]. These cells can harbor HIV for long periods, evading the immune system and ART [78]. The mechanisms by which HIV establishes and maintains latency in the CNS are complex and multi-layered [11]. Microglia are the resident immune cells of the CNS and are a major reservoir for latent HIV [79]. Latency is maintained through various cellular mechanisms, including the integration of HIV DNA into the host genome and suppression of viral gene expression by host cellular factors [80].

This integration of HIV into the genome of microglia is affected by the chromatin environment of the host, with HIV showing a tendency to integrate within transcriptionally active areas and topologically associated domains (TADs) that are rich in CCCTC-binding factor (CTCF), whereas CTCF removal impairs viral integration, highlighting the importance of genome organization in HIV-1 infection [81]. This was shown in vitro using a microglial cell model, in which HIV-1 insertions into introns of actively transcribed genes with integration site (IS) hotspots in genic and super-enhancers, which are characteristic of blood cells, are maintained.

Although the integration landscape of HIV has been well studied in peripheral blood CD4+ T cells, there is limited understanding of HIV integration sites in microglia in the brain. One study using archived frozen tissues mapped the HIV integration landscape in the brain during chronic HIV infection without suppressive ART and found that integration sites are enriched in actively expressed genes located in open chromatin regions in microglia [82]. The study shows that interactions between microglia and neurons are disrupted by HIV, and retroviral integration is associated with the 3D remodeling of the microglial genome during infection. Overall, HIV infection in the brain prompts IFN stimulation in microglia, induces chromatin reorganization into an active state, and alters the landscape of HIV integration [82,83].

The authors also characterized chromatin activity across several measures (active histone marks (H3K4me3 and H3K27ac) and chromatin accessibility (ATAC-seq)). Notably, HIV integration sites were found near accessible chromatin regions with active histone marks. It is worth mentioning that the HIV integration landscape here is different from the one observed in virally suppressed peripheral blood, and the authors did not find integration at highly enriched genes, like BACH2 or MKL2, or at centromeric regions [84]. This research presents a standardized methodology utilizing archived frozen tissues for single-nucleus transcriptome profiling to identify cell types and analyze their cellular pathways. This advancement expands our knowledge of HIV pathogenesis in the body beyond what has been seen within the restricted perspective offered by studies conducted on peripheral blood.

Epigenetic modifications, including histone modifications and DNA methylation, are key to maintaining HIV latency in the CNS [85]. Histone deacetylases (HDACs) and histone methyltransferases silence HIV in astrocytes and microglia. Class I HDACs and the lysine-specific histone methyltransferase SU(VAR)3-9 maintain HIV latency by modifying histones to create a repressive chromatin environment and prevent viral gene expression. Heterochromatin marks like H3K9me3 across the HIV genome in macrophages, plus activation marks like H3K9ac and H3K27ac at the LTR, suggest complex regulation of HIV transcription [86,87].

DNA methylation also contributes to HIV latency [88]. Latently infected cells have 5′-methylcytosine (5mC)-enriched HIV genomes, which are associated with transcriptional silencing. Five-hydroxymethylcytosine (5hmC) is enriched in actively transcribed HIV genomes in macrophages and CD4+ T cells, indicating dynamic regulation of viral gene expression through DNA methylation [87]. These epigenetic mechanisms maintain a reservoir of latent HIV in the CNS. Further, damaged neurons can trigger HIV reactivation, leading to a vicious cycle of neuroinflammation and neuronal damage [89]. Pro-inflammatory cytokines and signals from damaged neurons can inadvertently reactivate latent HIV, leading to intermittent cycles of viral reactivation and silencing, perpetuating chronic neuroinflammation and neuronal injury [90]. This ongoing inflammation and neuronal damage are the key to the development and progression of HAND, which results in cognitive impairment and neurodegeneration [1,91,92].

## 7. Latency and the “Kick and Kill” Strategy

The persistence of latent reservoirs is the main hurdle to an HIV cure [93]. ART is simply not sufficient to eradicate HIV reservoirs in the CNS, as the immune system fails to detect the presence of transcriptionally silent, latently infected cells, limiting recognition for elimination by immune-mediated clearance or direct viral cell lysis by viral production [94] (Table 1). The “kick and kill” approach is to reactivate (“kick”) latent HIV using latency-reversing agents (LRAs), such as HDAC inhibitors, and then clear (“kill”) the reactivated virus using ART or immune modulators [95]. In theory, reactivation of HIV with LRAs by targeting the latency mechanisms will lead to HIV RNA and viral protein production [96,97]. Subsequently, these reactivated cells will be recognized and killed by the host immune defense or viral cytolysis, as opposed to evading immune surveillance during latency [98].

The “kick and kill” approach, however, can cause adverse effects [99]. Reactivating latent HIV as part of the “kick and kill” strategy can cause increased inflammation and worsen neuroinflammation and HAND [100]. Robust HIV reactivation via LRAs will lead to encephalitis, neuronal damage, or neuronal loss because the LRA is non-specific and induces inflammation and viral toxicity, causing bystander cell death [101] (Table 1). Given the limited potential to replace these neurons, the “kick and kill” strategy might be harmful to brain function [102]. Moreover, better in vitro models, such as human cerebral organoid models, BMVECs, or in vivo animal studies, are needed to study HIV persistence in the CNS, including latency, latency reversal, and its impact on cell activation, viral production, immune activation, and safety.

In addition to the effects of viral reactivation in the CNS and its effects on neuron functionality, LRAs may also have direct neurotoxic consequences [103]. A significant concern regarding LRA is that they might not only trigger cells containing latent HIV but may also activate other dormant cells [104,105]. The current LRAs still do not exclusively activate HIV-infected cells, leading to immune activation and toxicity in nearby non-infected cells, particularly among CD4+ T cells, raising the concern that activation of non-infected cells in the CNS could also result in toxicity for these bystander cells [104,105].

Numerous in vitro experiments have been conducted using various LRAs that exhibited different outcomes in the culture model systems of microglia [94,106,107,108]. Studies have shown inconsistent results concerning the initiation of viral transcription. Variations between donors have also been suggested as a contributing factor to these discrepancies [107]. Current in vitro studies on LRAs using primary CNS cells have not demonstrated a clear, direct impact on decreasing the number of infected cells, even with viral reactivation [109]. However, it seems improbable that administering LRAs on their own will eradicate these cells, which is why LRAs should be combined with agents that promote cell killing. Below, we will explore adjunctive therapies. To the best of our knowledge, there are currently no investigations examining the effects of this approach on the CNS of people with HIV on ART [109].

In summary, the “kick and kill” approach is key to the HIV cure because it gets to the hidden reservoirs where the virus can persist despite ART [110]. However, because of potential side effects and the complexity of the CNS environment, it requires balancing latency reversal with targeted cell elimination, often requiring combination therapies and advanced delivery methods. Facilitating the “kill” is particularly challenging in the CNS due to the blood–brain barrier and the brain microenvironment. Multiple approaches have been investigated, such as focused ultrasound, receptor-mediated transport, exosomes, and nanoparticles, to enhance the delivery of therapeutic agents across the BBB and better target HIV reservoirs.

### 7.1. Focused Ultrasound

Focused ultrasound (FUS) with microbubbles is a way to deliver ART directly to the brain for the treatment of HAND (Table 1). This uses the ability of FUS to temporarily and non-invasively open the BBB at specific locations, allowing therapeutic agents to enter the CNS [111]. This has shown promise in preclinical models for delivering drugs to specific brain regions, potentially to treat HIV reservoirs in the CNS [112]. FUS works by using sound energy to make microbubbles in the bloodstream oscillate [113]. The oscillations create mechanical forces that open the tight junctions of the endothelial cells lining the BBB, increasing its permeability. This is very localized and can be controlled using imaging like MRI to target specific brain areas [112,114,115]. Studies have shown that FUS-mediated BBB opening can deliver various therapeutic agents, including antiretroviral drugs [116]. For example, FUS has been shown to increase the delivery of ARVs like tenofovir and abacavir to the brain, increasing their concentrations in the CNS and improving their efficacy [117,118]. This not only delivers more drugs but also reduces systemic side effects by requiring fewer drugs.

Moreover, FUS-mediated BBB disruption is safe and well-tolerated in preclinical models and early-phase clinical trials with reversible BBB opening and minimal side effects [119,120]. This could be promising for HAND management by delivering ART to CNS reservoirs and reducing viral persistence and neuroinflammation [121].

FUS, combined with microbubbles, can temporarily disrupt the BBB and alter the structure and function of microglia, leading to positive effects on the onset and progression of neurodegenerative diseases [122]. The oscillation pressure of FUS induces the expansion, contraction, or collapse of microbubbles administered intravenously, which creates a short-term increase in BBB permeability [123]. Differences in the composition of these microbubbles can impact the outcomes of FUS therapies. While most current clinical trials have primarily focused on brain regions without the use of intravenously delivered treatments, the combination of FUS and microbubbles has demonstrated the ability to enhance the delivery of therapeutic agents in patients with brain tumors, amyotrophic lateral sclerosis, and Parkinson’s disease [121,124,125]. Unlike intracranial injections, which cause tissue damage at each injection site due to the needle track, FUS can be utilized in any area of the brain in both rodents and humans without impacting surrounding regions, potentially enabling a targeted yet extensive distribution of therapeutics [126,127,128]. Current research is promising and indicates that microglia are found alongside misfolded proteins, facilitating their clearance. However, no research has yet investigated the nanometric effects of FUS or FUS combined with microbubbles on these cells to our knowledge. Microglia are in close communication with synaptic components and blood vessels within the CNS, raising the possibility that after modulating the BBB using FUS combined with microbubbles, the leakage of blood molecules and the release of endothelial chemokines could disrupt these interactions [129,130,131]. Nevertheless, considering the extensive functions of microglia in maintaining CNS health, it will be crucial to verify that both FUS and FUS paired with microbubbles can reduce the pathological activities of microglia while enhancing their homeostatic functions [132].

### 7.2. Receptor-Mediated Transport

Receptor-mediated transport is being explored as a way to deliver therapeutic agents across the BBB more efficiently, particularly using receptors like transferrin and insulin receptors to deliver ART and LRAs for HAND (Table 1).

The transferrin receptor is highly expressed on brain endothelial cells and allows iron-bound transferrin to cross the BBB [133]. This receptor has been targeted using transferrin-conjugated nanoparticles and immunoliposomes to deliver ART and other therapeutic agents to the CNS [134]. For example, transferrin receptor-targeted liposomes have shown increased uptake and transport of encapsulated drugs into the brain parenchyma, improving drug exposure and efficacy [135,136]. Transferrin receptor-targeting antibodies have also been engineered to act as molecular Trojan horses, ferrying therapeutic agents across the BBB via receptor-mediated endocytosis and transcytosis [137,138].

The insulin receptor is another target for receptor-mediated transport, using its natural role in transporting insulin across the BBB [139]. Monoclonal antibodies against the insulin receptor have been developed to deliver large molecule therapeutics, including ART and latency-reversing agents, into the brain [140]. These antibodies bind to the insulin receptor, triggering endocytosis and subsequent transcytosis, delivering the therapeutic payload into the CNS [137,138].

Receptor-mediated transport involves the binding of ligands (e.g., transferrin, insulin) or ligand-conjugated nanoparticles to their respective receptors on the endothelial cell surface [141]. This binding induces receptor-mediated endocytosis, followed by transcytosis across the endothelial cells and the release of the therapeutic agents into the brain parenchyma [142]. This has been shown to deliver ART and latency-reversing agents, potentially reducing viral reservoirs in the CNS and HAND [135,143,144].

### 7.3. Exosomes

Exosomes, which are natural vesicles secreted by cells, are also being investigated as a means to deliver drugs to CNS reservoirs for HAND [145]. Exosomes are 50–150 nm phospholipid bilayer vesicles that facilitate cell-to-cell communication and transport various biomolecules, including proteins, lipids, and nucleic acids [146]. They can cross the BBB, so they are attractive for CNS drug delivery [147,148]. Exosomes can be engineered to carry antiretroviral drugs (ARVs) and other therapeutic agents [149]. For example, exosomes from naive macrophages have been shown to deliver brain-derived neurotrophic factor (BDNF) across the BBB in mice models to inflamed brain regions [150,151]. This uses the natural homing ability of exosomes and their ability to interact with brain endothelial cells via integrin and lectin receptors. Exosomes can also be loaded with small-molecule drugs, proteins, and nucleic acids to target HIV reservoirs in the CNS. This has shown promise in reducing neuroinflammation and improving drug bioavailability in the brain [152,153]. Exosomes loaded with ARVs have shown enhanced delivery and efficacy in preclinical models of HAND [153]. Stem cell-derived exosomes are also being investigated for their neuroprotective and anti-inflammatory properties [154]. These exosomes can modulate neuroinflammation and promote neuronal repair, so a dual therapeutic approach by delivering ARVs and supporting CNS health [155,156].

Exosomes were frequently disregarded by scientists until research revealed their potential immunomodulatory properties [157,158]. Significant efforts have been made to study exosomes in relation to HIV within plasma and peripheral immune cells [159,160]. Overall, investigations have demonstrated that exosomes aid in the transport of viral proteins (such as Tat protein and Nef) and host proteins (including pro- and anti-inflammatory cytokines/chemokines and indicators of oxidative stress), thereby promoting viral spread [160,161]. Research has indicated that unspliced HIV-1 RNA sequences that code for Gag can be integrated into exosomes [162,163]. The exosome pathway within macrophages is crucial for the budding of HIV and promotes the infection of other cells by the virus [164]. The exosomal pathway in macrophages is crucial for HIV budding and aids in the infection of other cells by the virus [164,165,166]. Nevertheless, further research is needed to fully understand the role of exosomes in HIV-related neuroinflammation. Given their varied intracellular components, exosomes have the potential to provide diagnostic and predictive insights that could significantly improve the development of new therapeutic strategies [148].

### 7.4. Nanoparticles

Nanoparticles can help move antiretroviral drugs across the BBB [142]. For example, biodegradable brain-targeted polymeric nanoparticles have been developed to deliver optimized ARV therapy with antioxidant and anti-inflammatory neuroprotectants [162,167]. This has been shown to reduce neuroinflammation and oxidative stress in astrocytes and microglia in both in vitro and in vivo models [168].

Nanoparticles, especially lipid nanoparticles, have been engineered to move the drug across the BBB [169]. These nanoparticles can be designed to encapsulate ART drugs and release them at the site of infection [170]. Magnetic nanoparticles, for example, can be controlled by an external magnetic field to deliver drugs directly to the brain and are capable of crossing the BBB [171]. A study developed magnetic azidothymidine 5′-triphosphate (AZTTP) liposomal nanoformulations that could transmigrate across an in vitro BBB model under an external magnetic field [172]. This increased the permeability and uptake of the drug by monocytes and delivery to the brain.

Another in vitro study used magneto-electric nanoparticles to achieve the on-demand release of antiretroviral drugs. These nanoparticles, when exposed to a low alternating current magnetic field, could release the drug after crossing the BBB and maintain the functional and structural integrity of the drug [173]. The structural and functional integrity of the drug after the release was maintained via in vitro analysis. In summary, magnetic nanoparticles guided by external magnetic fields can be a solution for delivering antiretroviral therapy to the brain and overcoming the BBB and targeted delivery.

Similarly, hybrid magneto-plasmonic liposomes (MPLs) have been developed for multimodal image-guided and brain-targeted HIV treatment [174]. These MPLs encapsulating tenofovir disoproxil fumarate could transmigrate across the BBB in vitro and have a therapeutic effect against HIV-infected human microglia cells when guided by an external magnetic field [174].

Another approach is multifunctional nanotherapeutics that combine ARVs, LRAs, and drug-abuse antagonists. These nanoformulations have shown sustained release and effective BBB transmigration in vitro using primary human astrocytes, reducing HIV-1 infectivity and reactivating latent virus in primary CNS cells. This can potentially eliminate HIV reservoirs in the CNS and improve therapeutic adherence in drug-abusing populations [175].

Bioinspired ionic liquid-coated nanoparticles (IL-NPs) have also been developed to enhance CNS delivery. These IL-NPs can hitchhike on red blood cells, gain significant brain access, and preferentially accumulate in microglia. This has been shown to deliver ARVs like abacavir to the CNS effectively, retain antiviral efficacy, and reduce neuroinflammation [176]. The IL-NPs in this dual in vitro and vivo study had a specific affinity to red blood cells after intravascular injection and could traverse the BBB. Once in the brain, the IL-NPs evaded passive diffusion and were actively taken up by resident microglia for internalization, allowing access to neuro-HIV reservoirs. In vitro cell culture experiments showed significantly higher uptake of IL-NPs in neural cells compared to uncoated Poly(lactic-co-glycolic acid) (PLGA) nanoparticles. This showed a novel and efficient way of delivering nanoparticles to the brain by creating bioinspired ionic liquid coatings that allow them to stick to red blood cells after injection. Besides targeting specific tissues, the IL coating also promotes uptake by microglia in vivo. The researchers also loaded the antiretroviral drug abacavir, and in vitro tests show the drug is active, the IL-NPs are nontoxic to peripheral blood mononuclear cells (PBMCs), and the IL coating enhances the internalization of the nanoparticles into cells.

Macrophage-carried nanoformulated ARVs (nanoART) have been investigated to improve drug delivery to the brain. This uses the natural ability of macrophages to cross the BBB, transfer nanoART to brain endothelial cells, and increase drug levels in the CNS [177]. This has shown reduced viral load and decreased glial activation in animal models [178]. Sustained-release nanoART formulations, like those co-encapsulating tenofovir and vorinostat, have shown prolonged drug release and effective BBB transmigration [179]. These have shown good antiviral efficacy and potential to improve patient adherence to therapy [179]. PLGA has shown promise for HAND because it can modulate neuroinflammation and NLRP3 inflammasome activation [180]. PLGA nanoparticles are biocompatible and biodegradable, so they are suitable for drug delivery [180]. They can encapsulate therapeutic agents, stabilize them, increase their bioavailability, and deliver them to specific cells like macrophages and microglia, which are key players in the HAND pathogenesis [181,182]. PLGA nanoparticles can cross the blood–brain barrier and deliver drugs directly to the CNS [183]. This targeted delivery is crucial for HAND neuroinflammation, as conventional ART does not penetrate the CNS effectively [167,180]. Studies have shown that PLGA nanoparticles can reduce neuroinflammation by delivering anti-inflammatory and neuroprotective agents to the brain [168]. For example, through in vitro coculture studies in human microglia and astrocytes, as well as in vivo analysis with animal models, researchers found that PLGA-based nanocarriers can deliver antioxidants and anti-inflammatory drugs and reduce oxidative stress and inflammation in astrocytes and microglia [167]. This is particularly relevant for NLRP3 inflammasome activation, which is a major contributor to neuroinflammation in HAND.

However, PLGA degradation products can influence the immune response [184]. PLGA degrades into lactic acid and glycolic acid, which can create an acidic environment [184,185]. While some studies show that PLGA degradation can induce a pro-inflammatory response in macrophages and lead to M1 polarization and increased cytokine production, other studies suggest that PLGA nanoparticles can be engineered to minimize inflammatory effects and increase therapeutic efficacy [185,186].

In summary, PLGA-based nanocarriers offer a promising approach for HAND by increasing drug delivery to the CNS, reducing neuroinflammation, and potentially modulating NLRP3 inflammasome activation [180]. Although PLGA degradation can induce a pro-inflammatory response through the creation of an acidic environment, the inflammatory effect can be modulated by careful nanoparticle design [185]. PLGA is a promising material for targeted drug delivery in HAND treatment and has the potential to improve cognitive outcomes in HIV-infected individuals by addressing the underlying neuroinflammatory processes.

Another approach has been combining nanotechnology-based drug delivery systems like PEGylated solid lipid nanoparticles (SLNs) with the nose-to-brain pathway via intranasal administration or microneedle delivery [187]. This method can improve the stability and permeability of ART drugs across the nasal mucosa, bypassing the BBB and leading to higher drug concentrations in the brain [187,188]. This route minimizes systemic distribution, potentially reducing the side effects of systemic ART administration. Nanocrystals of antiretroviral drugs like rilpivirine and cabotegravir have been explored and showed promising results in therapeutic levels in the brain [189]. Intranasal administration can deliver targeted drug accumulation in the brain, making ART more effective in treating HAND [190]. However, nasal mucosa has low permeability and contains enzymes that can degrade ART drugs before they reach the CNS, reducing their efficacy and limiting their residence time and absorption [187,191]. Nose-to-brain delivery pathways have the potential to deliver ART into the CNS, but enzymatic degradation, mucociliary clearance, and low permeability are major challenges that need to be addressed for clinical translation.

## 8. Gene Editing and CRISPR/Cas9

Gene editing technologies, especially using clustered, regularly interspaced short palindromic repeats (CRISPR)/Cas9, can remove HIV from the genome of infected cells directly [192]. CRISPR/Cas9 works by using a guide RNA (gRNA) to direct the Cas9 nuclease to specific sequences in the HIV proviral DNA, where it introduces double-strand breaks. These breaks can lead to the excision of the integrated HIV genome, effectively removing the virus from the host cell’s DNA [192].

Recent studies have shown that CRISPR/Cas9 can target and excise HIV proviral DNA [193]. For example, Dash et al. showed that dual CRISPR therapies targeting CCR5 and HIV-1 proviral DNA eliminated replication-competent viruses in a significant proportion of humanized mice [194]. It was also demonstrated that synthetic gRNA/Cas9 ribonucleoproteins could inhibit HIV reactivation and replication in latently infected cells, showing that CRISPR/Cas9 can inactivate latent HIV reservoirs [195]. This has large implications for the “kick and kill” approach. By combining CRISPR/Cas9-mediated excision of HIV with LRAs that “kick” the virus out of latency, the virus is exposed and then can be eliminated in its reactivated form [196]. This dual approach could shrink the latent reservoir and achieve a functional cure for HIV [193]. In summary, CRISPR/Cas9 allows the removal of HIV from the genome of infected cells by introducing targeted double-strand breaks that excise the proviral DNA. This, with the “kick and kill” approach, could eliminate HIV and HAND (Table 1). 

While this works in a dish, getting CRISPR into the CNS is a big problem. One primary issue is the possibility of off-target effects, which could lead to significant gene mutations and chromosomal translocations [197,198]. Minimizing off-target effects is always a top priority in clinical contexts. Advances in viral vector delivery systems like adeno-associated virus (AAV) vectors may be the solution for targeting CNS reservoirs when using gene editing technologies like CRISPR/Cas9 to remove HIV from infected cells [199]. AAV vectors have many advantages for CNS targeting. They are non-pathogenic, low immunogenic, and can achieve long-term gene expression [200]. Specific AAV serotypes like AAV9 can cross the BBB and transduce CNS cells well [201]. For example, AAV9 vectors have been used to deliver CRISPR/Cas9 to the brain and achieve broad distribution and precise cleavage of integrated proviral DNA in various tissues, including the brain [201]. This has shown promise in reducing viral load in CNS reservoirs. Nonetheless, due to their limited packaging capacity, they can only carry a small exogenous gene, and re-administration of the same virus can provoke immune responses that diminish both delivery efficiency and gene expression [200,202] (Table 1).

Also, engineered AAV vectors like AAV9P1 have been developed to target astrocytes specifically. These vectors have been used to deliver CRISPR/Cas9 to latently HIV-1 infected astrocytes and resulted in reduced reactivation of proviruses and mutations/deletions in key HIV-1 transcriptional control regions [203]. This shows that AAV vectors can target and inactivate HIV in CNS reservoirs. Also, AAV2g9 have been engineered to have robust neuronal transduction with minimal off-target effects, so they are suitable for CNS-targeted gene therapy [57]. AAV vectors are a potential way to deliver CRISPR/Cas9 to CNS reservoirs to eradicate HIV in the brain by targeting and removing latent HIV from infected cells [201,203,204].

## 9. Conclusions

ART is effective at suppressing viral replication in the blood, but the establishment of latent viral reservoirs in CNS cells, disruption of the BBB, and chronic neuroinflammation continue to drive HIV-associated neurocognitive disorders. Latent reservoirs are established by complex interactions between viral factors, neuroinflammation, and BBB disruption and persist despite systemic viral suppression. Understanding the molecular and cellular mechanisms of HIV persistence in the CNS has led to many advances in therapeutics. Key mechanisms of HIV persistence in the brain, including viral entry through compromised BBB, monocyte trafficking, and activation of microglia and astrocytes, feed into the cycle of neuroinflammation and neuronal damage. Epigenetic changes that maintain HIV latency in CNS resident cells make it unobtainable to clear the virus even with current ART regimens.

HIV latency and the inflammation that worsens CNS damage need to be targeted to develop therapies that can clear these reservoirs. New approaches like the “kick and kill” strategy, CRISPR/Cas9 gene editing, and advanced drug delivery systems are emerging as potential ways to directly target and eliminate latent HIV. Targeting epigenetic regulators of latency and improving BBB penetration through receptor-mediated transport and other methods are the next steps. Advances in drug delivery technologies like nanoparticles, exosomes, and focused ultrasound can help overcome the challenge of delivering therapeutics across the BBB to reach CNS reservoirs. The assessment of HIV cure approaches in the CNS, including the “kick and kill” method, is still difficult, and there are multiple limitations concerning the effectiveness and safety of reactivating the viral reservoir with LRAs in the CNS.

Hence, gaining a deeper understanding of the mechanisms underlying HIV latency in the CNS and the possible effects of latency reversal on neuropathogenesis is crucial. Enhancements in the delivery of LRAs are being investigated, such as the creation of nanoparticles that can specifically target HIV-infected cells that are in a latent state within the CNS. The combination of LRAs with agents that induce apoptosis may also enhance the effectiveness and safety of the “kick and kill” approach by making cells more susceptible to death soon after HIV reactivation, thus reducing ongoing viral replication and limiting damage to surrounding non-infected cells. Ideally, these strategies will increase both the effectiveness and specificity of the drug compounds directed at HIV-infected cells, aiming to increase the eradication of these cells while minimizing the removal of non-infected brain cells.

Moreover, improved in vitro models, such as human cerebral organoids, or in vivo studies in animal models are necessary to explore the mechanisms of HIV persistence in the CNS, including latency, the reversal of latency, and its effects on cell activation, viral replication, immune system activation, and overall safety. Vigorous HIV reactivation through LRAs is likely to result in encephalitis, neuronal damage, or the loss of neurons due to the generally non-specific stimulation from LRAs, inflammation, and viral toxicity that leads to the death of bystander cells. Given the limited ability to regenerate these neurons, the “kick and kill” strategy might negatively impact brain function. For this reason, it is essential to track HIV RNA levels in CSF and monitor specific CSF parameters during clinical trials to evaluate the effects and safety of the “kick and kill” strategy.

While we have made progress in understanding HIV, BBB, and neuroinflammation, more research is needed to turn it into treatment. Combination therapies that address HIV latency, neuroinflammation, and BBB disruption, along with new CNS drug delivery methods, are the way to improve the long-term outcomes of people living with HIV. Ultimately, clearing HIV from the CNS is key to a functional cure, and more research will continue to lead to better and more comprehensive treatment for HAND.

## Figures and Tables

**Table 1:** Therapeutic strategies: mechanisms, drug delivery systems, advantages, and limitations.

Therapeutic Strategy	Mechanism of Action	Drug Delivery System	Advantages	Limitations
Antiretroviral Therapy (ART)	Suppresses viral replication, reduces systemic viral load	Oral, injectable, nanoparticle formulations	Established efficacy, reduces viral reservoirs	Limited BBB penetration, cannot target latent reservoirs
Latency-Reversing Agents (LRAs)	Reactivates latent HIV for immune clearance	Small molecules, histone deacetylase inhibitors (HDACi)	Potential to purge latent HIV reservoirs	Potential off-target effects, inflammation risk
Gene Editing (CRISPR/Cas9)	Excises integrated HIV DNA from host genome	AAV-based delivery, Lipid Nanoparticles	Permanent virus removal, potential cure	Delivery challenges, ethical and safety concerns
Neuroprotective Agents	Protects neurons from damage and apoptosis	Peptides, growth factors, small molecules	Preserves neuronal integrity, reduces oxidative stress	Limited BBB permeability, needs improved formulations
Anti-Inflammatory Drugs	Reduces neuroinflammation and cytokine release	NSAIDs, corticosteroids, IL-1β inhibitors	Targets neuroinflammation, prevents progression of HAND	Potential systemic side effects, incomplete neuroprotection
Monocyte/Microglia Modulators	Modulates immune cell trafficking to the CNS	CCR5 inhibitors, CCL2 modulators	Reduces immune cell-mediated neuroinflammation	Incomplete efficacy in reducing viral reservoirs
Focused Ultrasound (FUS)	Temporarily disrupts the BBB for targeted drug delivery	Microbubble-assisted ART delivery	Non-invasive, localized CNS drug delivery	Risk of non-specific BBB opening, transient effects
Receptor-Mediated Transport (RMT)	Uses transferrin/insulin receptors for BBB penetration	Nanocarriers conjugated to transferrin/insulin	Efficient brain penetration with minimal systemic toxicity	Receptor saturation limits delivery capacity
Exosome-Based Delivery	Natural vesicles for targeted drug delivery to CNS	Exosome-loaded ART and anti-inflammatory agents	Biocompatible, targeted drug delivery	Low drug loading capacity, limited scalability
Nanoparticle-Based Drug Delivery	Enhances drug penetration across BBB, sustained release	Polymeric, lipid, magnetic, and hybrid nanoparticles	Prolonged drug release, targeted CNS penetration	Potential toxicity, clearance limitations

## Data Availability

Data available in references.
